# Rotational vs. laser atherectomy in Chinese CTO-PCI: lesion-specific efficacy with comparable midterm safety

**DOI:** 10.3389/fcvm.2025.1650595

**Published:** 2025-10-31

**Authors:** Liansheng Chen, Zehan Huang, Quanmin Wu, Huiliang Deng, Meiping Huang, Yiqi Xu, Jinkun Wei, Yong Liu, Nianjin Xie, Yuming Huang

**Affiliations:** ^1^ Department of Catheterization Lab, Guangdong Cardiovascular Institute, Guangdong Provincial People's Hospital (Guangdong Academy of Medical Sciences), Southern Medical University, Guangzhou, China; ^2^ Pediatrics, Nanfang Hospital, Southern Medical University, Guangzhou, China; ^3^ Department of Cardiovascular, Xiaolan People's Hospital of ZhongShan (The Fifth People's Hospital of ZhongShan), Zhongshan, China; ^4^ Department of Cardiovascular, Guangdong Provincial People's Hospital Ganzhou Hospital, Ganzhou, China

**Keywords:** chronic total occlusion, percutaneous coronary intervention, in-hospital outcomes, long-term outcomes, excimer laser coronary atherectomy, rotational atherectomy, procedural costs

## Abstract

**Background:**

Chronic total occlusion (CTO) percutaneous coronary intervention (PCI) often requires plaque modification for device delivery. While rotational atherectomy (RA) and excimer laser coronary atherectomy (ELCA) are established adjuncts, their comparative efficacy and safety remain underexplored in Chinese populations.

**Methods:**

This single-center retrospective study included 75 consecutive CTO-PCI patients treated with ELCA (*n* = 25) or RA (*n* = 50). Procedural success, complications, and major adverse cardiovascular and cerebrovascular events (MACCE) were analyzed over a median 17.5-month follow-up. Multivariable Cox regression adjusted for calcification severity, lesion length, ISR-CTO, and diabetes mellitus.

**Results:**

RA was preferred for moderate/severe calcification (76% vs. 48%, *p* = 0.020), while ELCA dominated in ISR-CTO (20% vs. 2%, *p* = 0.024) and lesions >20 mm (56% vs. 30%, *p* = 0.044). Procedural success was comparable (RA 90% vs. ELCA 84%, *p* = 0.706). Procedure-related complications differed: RA had two coronary perforations (4% vs. 0%, *p* = 0.130), whereas ELCA showed a trend toward more transient slow/no-reflow (12% vs. 0%, *p* = 0.061). MACCE rates remained similar (19% vs. 13.3%, *p* = 0.815; adjusted HR 1.53, 95% CI 0.35–6.65, *p* = 0.569). Both techniques exhibited comparable procedural duration and radiation exposure (all *p* > 0.05). ELCA incurred higher total costs (US11,147 vs. 9,267, *p* = 0.007), driven by laser catheter expenses; however, procedural costs became comparable after excluding catheter-related expenditures (*p* = 0.210).

**Conclusion:**

In Chinese CTO-PCI, ELCA and RA demonstrate lesion-specific utility—ELCA for ISR-CTO and long lesions, RA for calcified lesions—with comparable midterm safety. Procedural costs of ELCA and RA were equivalent in Device-excluded costs analysis.

## Introduction

Chronic total occlusion (CTO) of the coronary arteries remains one of the most challenging lesion types in percutaneous coronary intervention (PCI). Successful CTO recanalization significantly improves myocardial perfusion, alleviates angina symptoms, enhances left ventricular function (1). However, CTO-PCI is often complicated by device delivery failure, particularly when balloons or microcatheters cannot cross the lesion despite successful guidewire passage, primarily due to severe calcification or non-compliant plaques (2, 3). This limitation underscores the critical need for effective plaque modification strategies.

Rotational atherectomy (RA) has been established as an effective adjunctive technique when conventional devices fail to cross or dilate CTO lesions (4). However, data from the 18th Oriental Congress of Cardiology (OCC-WCC 2024) indicate that RA utilization in China remains remarkably low (0.9%), significantly below Western adoption rates. RA requires dedicated RotaWire™ exchange, which becomes unfeasible if a microcatheter cannot cross the lesion, thus limiting its applicability. In recent years, excimer laser coronary atherectomy (ELCA) has emerged as a promising alternative, enabling plaque modification without guidewire exchange—even when microcatheters fail to traverse the occlusion (5). Despite these advantages, ELCA adoption in mainland China remains limited compared to RA. Both techniques may still fail in cases of extreme calcification or when the true lumen cannot be confirmed post-recanalization, carrying elevated procedural risks (6, 7).

Notably, direct comparisons between ELCA and RA in CTO-PCI are scarce in China. This study therefore aims to evaluate and compare procedural success rates, complications, and the incidence of major adverse cardiovascular and cerebrovascular events (MACCE) during follow-up between ELCA and RA for CTO treatment, providing critical insights for contemporary practice.

## Methods

### Study population

**Figure 1:**
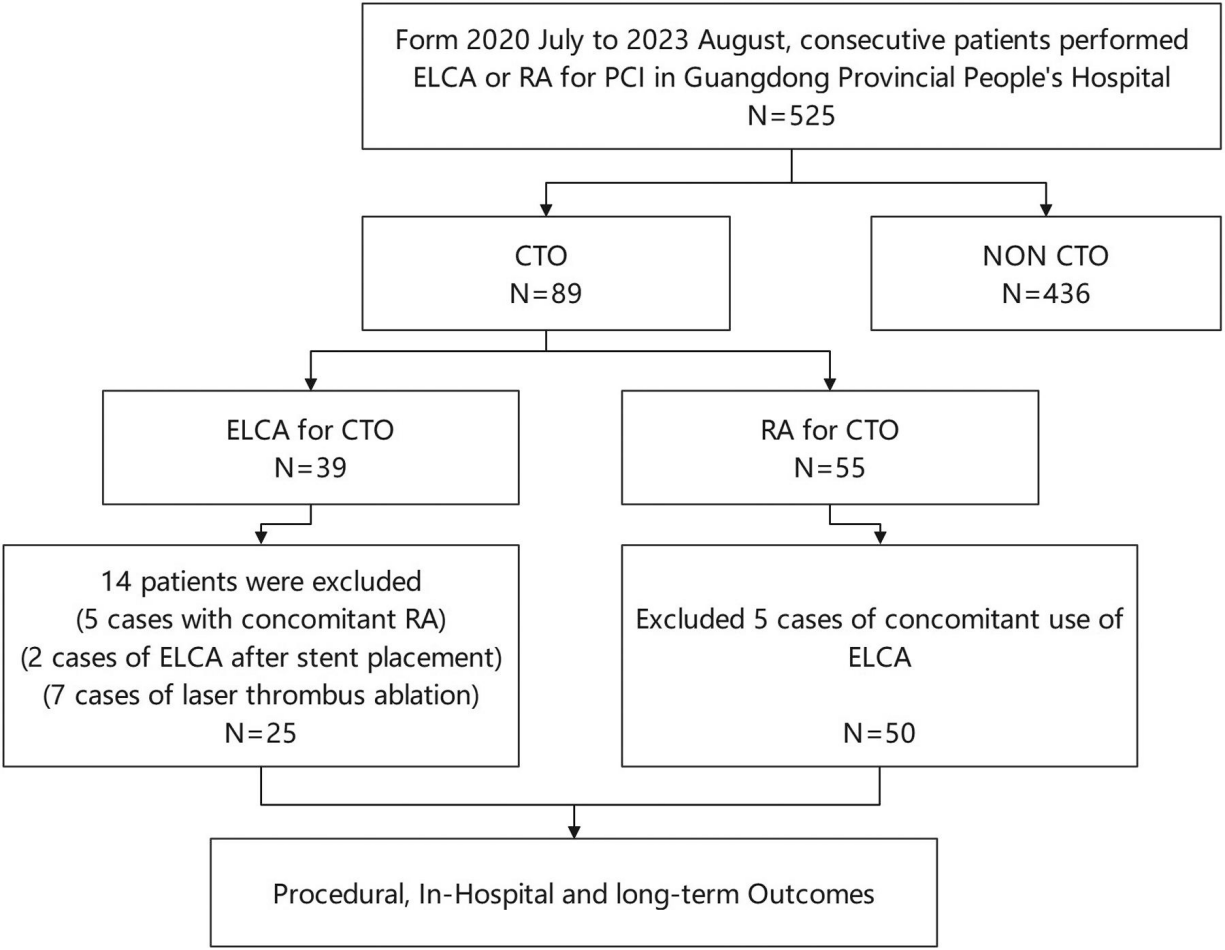
Flow chart of the study population.

**Figure 2:**
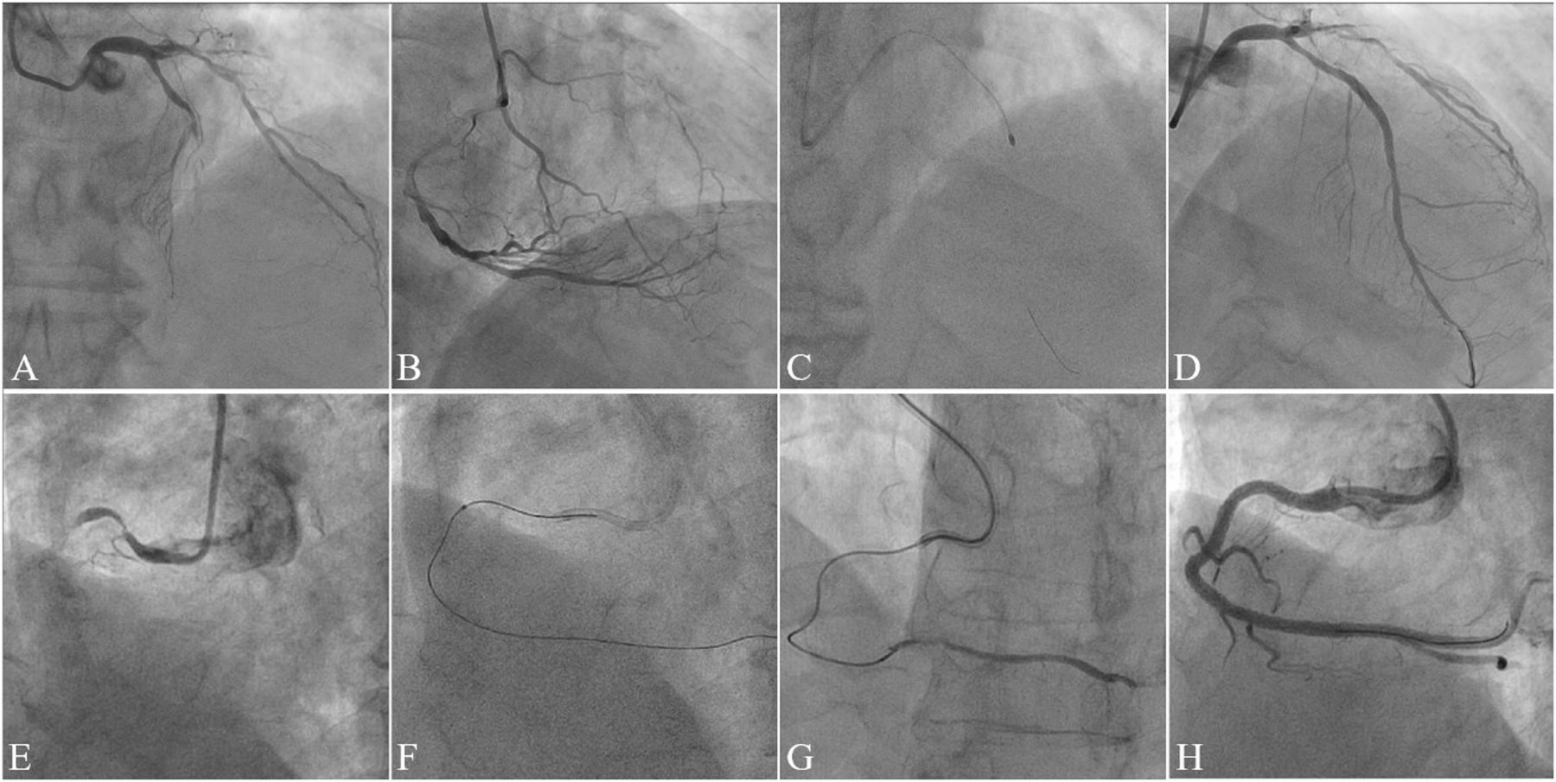
Case illustrations of CTO-PCI with rotational atherectomy (RA) and excimer laser coronary atherectomy (ELCA). Case 1. **(A)** Preprocedural angiography demonstrating mid-to-distal left anterior descending artery (LAD) occlusion (TIMI 0 flow); **(B)** Right coronary artery (RCA) angiography showing collateral circulation supplying the distal LAD; **(C)** Rotational atherectomy (RA) performed after failed antegrade wiring and balloon uncrossability; **(D)** Final angiography post-stenting demonstrating restored patency (TIMI 3 flow). Case 2. **(E)** Preprocedural angiography revealing proximal RCA occlusion (TIMI 0 flow); **(F)** Excimer laser coronary atherectomy (ELCA) initiated after failed antegrade microcatheter advancement; **(G)** Post-ELCA microcatheter advancement to the distal RCA true lumen (confirmed by contrast injection); **(H)** Final angiography post-stenting showing optimal vessel reconstruction.

This study analyzed medical records of consecutive patients who underwent ELCA or RA at Guangdong Provincial People's Hospital (July 2020–August 2023). The choice between ELCA and RA was made at the operator's discretion, based on a comprehensive assessment of specific lesion characteristics. From 525 initially screened procedures, we identified 89 patients with CTO exhibiting device-uncrossable or balloon-undilatable lesions. Ultimately, 75 eligible patients were included in this study. The inclusion and exclusion process of the study subjects was detailed in Figure 1. The inclusion criteria for this study strictly adhered to the following points: (1) age ≥18 years; (2) CTO-PCI indications including angina symptoms and/or objective evidence of reversible myocardial ischemia (perfusion imaging/stress testing). The exclusion criteria were: (1) age >85years or high bleeding risk contraindicating surgery; (2) contraindications to dual antiplatelet therapy; (3) history of radiation skin injury; (4) severe renal impairment without dialysis commitment. This study has been strictly approved by the Ethics Committee of Guangdong Provincial People's Hospital (approval number: KY2023-716-01), and in accordance with regulations, participants are not required to sign an informed consent form. Figure 2 presents representative cases of CTO-PCI, one performed with ELCA and the other with RA.

### Definitions

A CTO was defined as a coronary artery occlusion with Thrombolysis in Myocardial Infarction (TIMI) flow grade 0 persisting for >3 months, as determined by symptom onset, documented myocardial infarction (MI) in the territory, or prior angiographic evidence. Lesion complexity was assessed using the Japanese CTO (J-CTO) score, with collateral circulation graded by the Werner classification (8). Moderate/severe calcification required clearly visible high-density shadows on angiography during both systolic and diastolic phases (9), while significant tortuosity was defined as ≥2 bends >70° or one bend >90° (10). The retrograde technique involved using specialized guidewires and microcatheters to approach the occlusion distally through collateral channels. Procedure time was measured from catheterization laboratory entry to exit. Technical success required successful stent/balloon delivery with <30% residual stenosis and TIMI 3 flow; procedural success additionally mandated absence of in-hospital MACCE, including death, periprocedural MI [Fourth Universal Definition (11)], urgent revascularization, pericardial drainage, surgical tamponade, or stroke. Follow-up MACCE comprised cardiovascular death, non-fatal MI, ischemia-driven revascularization, and stroke, with stent thrombosis defined by Academic Research Consortium criteria (12).

### Excimer laser coronary angioplasty

ELCA was performed using the CVX-300 excimer laser system (Spectranetics Corporation, Colorado Springs, CO, USA), which operates at 308 nm wavelength with 135 ns pulse width and 165 mJ/pulse energy output (13). The Vitesse CTM laser atherectomy catheter (diameter range: 0.9–1.7 mm) was selected based on angiography or intracoronary imaging, maintaining a catheter-to-vessel diameter ratio of 0.67 ± 0.21. All procedures employed saline-flush technique: (1) pre-irrigation with 0.9% saline to clear contrast media; (2) bolus injection of 5–10 ml saline before each laser activation; (3) continuous saline infusion (1 ml/s) during lasing. The catheter was advanced at 0.5–1 mm/s with pulsed activation (≤10 s/pulse, ≥5 s intervals) at energy densities of 35–80 mJ/mm² and frequencies of 35–80 Hz.

### Rotational atherectomy

RA was performed using the Rotablator system (Boston Scientific, Marlborough, MA, USA). Burr selection was based on vessel diameter and plaque burden, with diameters ranging from 1.25 to 1.75 mm, and the maximum burr size was confirmed by angiography or intracoronary imaging to ensure adequate debulking. The rotational speed was maintained at 170,000–180,000 rpm, increased to 200,000 rpm for severely calcified lesions when needed. Following RA, the rotational guidewire was exchanged for a working guidewire, followed by balloon angioplasty or stent implantation to optimize angiographic results and minimize residual stenosis.

### Follow-up

Follow-up data were collected through outpatient visits, hospital admissions, or telephone interviews, with clinical documentation during each encounter. The primary endpoint was MACCE, defined as a composite of cardiovascular mortality, non-fatal MI, ischemia-driven target vessel revascularization (TVR), and stroke. All procedures followed the Declaration of Helsinki principles.

### Statistical analysis

Continuous variables with normal distribution were presented as mean ± standard deviation and compared using Student's *t*-test, while non-normally distributed variables were expressed as median (interquartile range) and analyzed with Mann–Whitney *U*-test. Categorical variables were reported as frequencies (%) and compared using *χ*² or Fisher's exact tests. Time-to-event outcomes were assessed using Kaplan–Meier methodology (log-rank test) and Cox proportional hazards models with adjustment for key clinical confounders. A two-tailed *p* < 0.05 was considered statistically significant. All analyses were conducted using MedCalc (v14.12.0, Ostend, Belgium).

## Results

### Baseline clinical characteristics

Seventy-five consecutive patients undergoing PCI for CTO were treated with either ELCA (*n* = 25) or RA (*n* = 50). Baseline characteristics were well-balanced between groups (Table 1), with no significant differences in mean age (ELCA: 64.76 ± 9.42 vs. RA: 67.22 ± 8.58 years), comorbidities (hypertension: 68% vs. 72%; dyslipidemia: 52% vs. 58%; diabetes mellitus: 36% vs. 2%), smoking status (28% vs. 34%), or prior revascularization [PCI: 24% vs. 30%; coronary artery bypass grafting (CABG): 8% vs. 6%] (all *p* > 0.05). Serum creatinine (1.12 ± 0.31 vs. 1.08 ± 0.28 mg/dl) and left ventricular ejection fraction (LVEF) (52.4 ± 8.7% vs. 53.1 ± 9.2%) were also comparable.

**Table 1:** Demographic data in patients treated with ELCA group and RA group.

Variable	ELCA group (ELCA for CTO), *N* = 25	RA group (Rota for CTO), *N* = 50	*p*-value*
Male, *n* (%)	22 (88)	39 (78)	0.463
Age (years)	64.76 ± 9.42	67.22 ± 8.58	0.261
BMI (kg/m²)	23.25 ± 2.29	24.44 ± 3.30	0.161
Hypertension, *n* (%)	10 (40)	16 (32)	0.493
Diabetes mellitus, *n* (%)	15 (60)	23 (46)	0.253
Dyslipidemia, *n* (%)	5 (20)	5 (10)	0.401
Current smoker, *n* (%)	12 (48)	16 (32)	0.177
Family History of CAD, *n* (%)	0 (0)	4 (8)	0.364
Prior MI, *n* (%)	6 (24)	15 (30)	0.585
Prior PCI, *n* (%)	9 (36)	14 (28)	0.479
Prior CABG, *n* (%)	0 (0)	1 (2)	>0.999
Serum creatinine, µmol/L	85.60 (71.00,112.26)	79.68 (66.39,108.48)	0.551
Baseline LVEF (%)	54.00 ± 14.25	54.67 ± 11.99	0.844
HDL-chol, mg/dl	0.96 ± 0.26	0.95 ± 0.19	0.788
LDL-chol, mg/dl	2.43 ± 0.95	2.33 ± 0.79	0.620

BMI, body mass index; MI, myocardial infarction; PCI, percutaneous coronary intervention; CABG, coronary artery bypass grafting; LVEF, left ventricular ejection fraction; HDL, high-density lipoprotein; LDL, low-density lipoprotein; ELCA, excimer laser coronary angioplasty; RA, rotational atherectomy; CTO, chronic total occlusion.

* *p* < 0.05 is considered significant.

### Angiographic characteristics

Angiographic characteristics of coronary artery lesions are compared in Table 2. Significant differences were observed between the groups in several parameters. The proportion of lesions longer than 20 mm was significantly higher in the ELCA group (56% vs. 30%, *p* = 0.044). CTO caused by in-stent restenosis (ISR-CTO) were more prevalent in the ELCA group (20% vs. 2%, *p* = 0.024). Most notably, moderate/severe calcification was significantly more frequent in the RA group (76% vs. 48%, *p* = 0.020). Lesion complexity assessed by J-CTO score was comparable between groups (1.88 ± 1.2 vs. 1.86 ± 0.9, *p* = 0.942), as was moderate/severe tortuosity (28% vs. 12%, *p* = 0.161). Although the distribution of CTO target vessels did not differ significantly between groups, the RA group showed a numerically higher rate of prior failed CTO-PCI (20% vs. 4%, *p* = 0.134). Both groups had high and comparable rates of collateral circulation and multivessel disease. No significant differences were observed in the distribution of target vessels or lesion locations between the two groups.

**Table 2:** Angiographic characteristics.

Variable	ELCA group (ELCA for CTO), *N* = 25	RA Group (Rota for CTO), *N* = 50	*p-value* *
Vessels			0.337
LAD, *n* (%)	11 (44)	30 (60)	
LCX, *n* (%)	1 (4)	3 (6)	
RCA, *n* (%)	13 (52)	17 (34)	
Location			0.475
Ostial, *n* (%)	2 (8)	3 (6)	
Proximal, *n* (%)	14 (56)	20 (40)	
Middle, *n* (%)	9 (36)	26 (52)	
Distal, *n* (%)	0 (0)	1 (2)	
Multivessel, *n* (%)	17 (68)	32 (64)	0.731
Blunt stump, *n* (%)	9 (36)	19 (38)	>0.999
Collateral circulation, *n* (%)	21 (84)	41 (82)	>0.999
ISR-CTO, *n* (%)	5 (20)	1 (2)	0.024
Lesion length >20 mm, *n* (%)	14 (56)	15 (30)	0.044
Prior failed CTO-PCI, *n* (%)	1 (4)	10 (20)	0.134
Moderate/severe calcification, *n* (%)	12 (48)	38 (76)	0.020
Moderate/severe tortuosity, *n* (%)	7 (28)	6 (12)	0.161
J-CTO score	1.88 ± 1.2	1.86 ± 0.9	0.942

LAD, left anterior descending artery; LCX, left circumflex artery; RCA, right coronary artery; CTO, chronic total occlusion; PCI, percutaneous coronary intervention; ISR, in-stent restenosis; ELCA, excimer laser coronary angioplasty; RA, rotational atherectomy.

* *p* < 0.05 is considered significant.

### Procedural characteristics

Table 3 summarizes the procedural characteristics of both groups. During guidewire crossing of occluded lesions, severe plaque burden frequently prevented device advancement (microcatheters or balloons), with significantly higher device-crossing failure rates observed in the ELCA group compared to the RA group prior to atherectomy (72% vs. 48%, *p* = 0.048). The two groups demonstrated comparable numbers of balloons used for pre-dilation (5.76 ± 3.11 vs. 5.90 ± 2.48, *p* = 0.846) and achieved similar maximum pre-dilation pressures (15.52 ± 3.4 atm vs. 15.73 ± 4.3 atm, *p* = 0.845). Post-stent dilation pressures were also equivalent (19.10 ± 4.56 atm vs. 21.24 ± 3.69 atm, *p* = 0.053). Intravascular ultrasound (IVUS) or optical coherence tomography (OCT) guidance was frequently used in both groups, with comparable utilization rates between the ELCA and RA groups (44% vs. 40%, *p* = 0.740). Both groups required implantation of comparable numbers of stents (2.5 ± 0.9 vs. 2.3 ± 0.8) with similar total stent lengths (72.8 ± 35.6 vs. 68.4 ± 26.4 mm; all *p* > 0.05). No significant differences were observed in technical success (88% vs. 96%, *p* = 0.413) or procedural success rates (84% vs. 90%, *p* = 0.706) between the two groups. Fluoroscopy time, total procedure duration, and radiation dose were marginally higher in the ELCA group compared to the RA group, although without statistical significance. Total procedural costs were significantly higher in the ELCA group (US$11,147.48 ± 2,334.89 vs. US$9,266.65 ± 2,711.36; *p* = 0.007), while device-excluded costs showed no significant difference (US$6,433.19 ± 2,334.89 vs. US$7,291.65 ± 2,711.36; *p* = 0.210).

**Table 3:** Lesion characteristics and procedure details in patients treated with ELCA group and RA group.

Variable	ELCA group (ELCA for CTO), *N* = 25	RA group (Rota for CTO), *N* = 50	*p-value* *
Access site			0.729
Single radial access, *n* (%)	16 (64)	34 (68)	
Any femoral access, *n* (%)	9 (36)	16 (32)	
Guide size			0.509
6F, *n* (%)	12 (48)	20 (40)	
7F, *n* (%)	13 (52)	30 (60)	
Failed to cross, *n* (%)	18 (72)	24 (48)	0.048
Failed to expand, *n* (%)	1 (4)	2 (4)	>0.999
TIMI >Ⅱ flow post PCI, *n* (%)	22 (88)	49 (98)	0.203
IVUS/OCT used, *n* (%)	11 (44)	20 (40)	0.740
Largest burr used (mm)			
1.25, *n* (%)	–	22 (44)	
1.50, *n* (%)	–	27 (54)	
1.70, *n* (%)	–	1 (2)	
Largest laser catheter used (mm)			
0.9, *n* (%)	15 (60)	–	
1.4, *n* (%)	5 (20)	–	–
1.7, *n* (%)	5 (20)	–	
Cutting balloons or double-coated balloons, *n* (%)	9 (36)	22 (44)	0.507
Total fluoroscopy time (min)	60.47 ± 32.30	54.36 ± 36.40	0.547
Total procedure time (min)	153.16 ± 58.75	139.30 ± 62.48	0.359
AK (Gy)	2.65 (1.66,3.04)	2.06 (1.15,3.40)	0.206
Mean stent numbers	2.53 ± 0.94	2.28 ± 0.78	0.296
Total stent length (mm)	72.76 ± 35.55	68.39 ± 26.38	0.598
Procedural costs (USD)	11,147.48 ± 2,334.89	9,266.65 ± 2,711.36	0.007
Device-excluded costs (USD)	6,433.19 ± 2,334.89	7,291.65 ± 2,711.36	0.210
Number of balloons	5.76 ± 3.11	5.90 ± 2.48	0.846
Balloon diameter pre-dilatation, mm	2.20 ± 0.37	2.44 ± 0.37	0.011
Maximum inflation pressure pre-dilatation, atm	15.52 ± 3.40	15.73 ± 4.30	0.845
Balloon diameter post-dilatation, mm	3.46 ± 0.56	3.36 ± 0.56	0.512
Maximum inflation pressure pro-dilatation, atm	19.10 ± 4.56	21.24 ± 3.69	0.053
CTO technique			0.203
Antegrade, *n* (%)	22 (88)	49 (98)	
Retrograde, *n* (%)	3 (12)	1 (2)	
Technical success, *n* (%)	22 (88)	48 (96%)	0.413
Procedural success, *n* (%)	21 (84)	45 (90%)	0.706
Contrast volume (ml)	138.40 ± 50.80	137.80 ± 49.99	0.961

TIMI, thrombolysis in myocardial infarction; IVUS, intravascular ultrasound; OCT, optical coherence tomography; AK, air kerma; PTCA, percutaneous transluminal coronary angioplasty; PCI, percutaneous coronary intervention; CTO, chronic total occlusion; ELCA, excimer laser coronary angioplasty; RA, rotational atherectomy.

* *p* < 0.05 is considered significant.

### Procedural complications and in-hospital outcomes

Table 4 summarizes procedural complications and in-hospital outcomes. No significant difference in major procedural complications was observed between groups (ELCA 24% vs. RA 16%, *p* = 0.600). However, the ELCA group showed a higher incidence of acute slow/no-reflow phenomena (12% vs. 0%, *p* = 0.061), while coronary perforation with cardiac tamponade occurred exclusively in the RA group (4% vs. 0%, *p* = 0.550), including one case requiring emergent pericardiocentesis followed by coil embolization. Two in-hospital deaths (multi-organ failure) occurred in the ELCA group, both adjudicated as unrelated to the laser procedure. No in-hospital mortality was observed in the RA group, though one patient required implantable cardioverter-defibrillator (ICD) implantation for recurrent ventricular tachycardia before discharge.

**Table 4:** Incidence of in-procedure complications in patients treated with ELCA group and RA group.

Variable	ELCA group (ELCA for CTO), *N* = 25	RA group (Rota for CTO), *N* = 50	*p-value* *
Procedural complications, *n* (%)	6 (24)	8 (16)	0.600
Acute slow/no reflow, *n* (%)	3 (12)	0 (0)	0.061
Coronary artery perforation, *n* (%)	0 (0)	2 (4)	0.550
Acute heart failure, *n* (%)	0 (0)	0 (0)	–
Ventricular tachycardia, *n* (%)	0 (0)	1 (2)	>0.999
Emergent CABG, *n* (%)	0 (0)	0 (0)	–
Covered stent implantation, *n* (%)	0 (0)	0 (0)	–
Stent thrombosis, *n* (%)	0 (0)	0 (0)	–
In-hospital MACCE, *n* (%)	2 (8)	1 (2)	0.532
Death, *n* (%)	2 (8)	0 (0)	0.108
Periprocedural MI, *n* (%)	0 (0)	0 (0)	–
TVR, *n* (%)	0 (0)	0 (0)	–
Stroke, *n* (%)	1 (4)	1 (2)	>0.999
Cardiac tamponade, *n* (%)	0 (0)	2 (4)	0.550
Pericardiocentesis, *n* (%)	0(0)	1(2)	>0.999

CABG, coronary artery bypass grafting; MACCE, major adverse cardiovascular and cerebrovascular events; MI, myocardial infarction; ELCA, excimer laser coronary angioplasty; RA, rotational atherectomy; CTO, chronic total occlusion; TVR, target vessel revascularization.

* *p* < 0.05 is considered significant.

### Clinical outcomes during follow-up

The clinical outcomes during follow-up are presented in Table 5. Patients with procedural failure were excluded from analysis, with the remaining cohort (follow-up rate: 88%) followed for a median of 16 months (IQR 12–26) in the ELCA group vs. 18 months (IQR 13.5–25.5) in the RA group. No significant between-group differences were observed in MACCE, cardiac rehospitalization, or all-cause mortality (all *p* > 0.05). No cardiac deaths occurred in either group. Non-cardiac deaths in the RA group (*n* = 3) included acute alcohol poisoning, complications of end-stage diabetes mellitus, and one undetermined cause. In the ELCA group, two stroke cases (lacunar infarction and brainstem infarction) were managed medically without residual disability. The RA group had one case of gastrointestinal bleeding (1-month post-PCI), potentially attributable to antiplatelet therapy, which resolved with hospitalization.

**Table 5:** Clinical outcomes on follow-up.

Variable	ELCA group (ELCA for CTO), *N* = 21	RA group (Rota for CTO), *N* = 45	*p-value* *
MACCE, *n* (%)	4 (19)	6 (13.3)	0.815
Cardiac death, *n* (%)	0 (0)	0 (0)	–
TVR, *n* (%)	2 (9.5)	5 (11.1)	>0.999
MI, *n* (%)	0 (0)	1 (2.2)	>0.999
Stroke, *n* (%)	2 (9.5)	0 (0)	0.098
Heart failure, *n* (%)	1 (4.8)	3 (6.7)	>0.999
Angina, *n* (%)	2 (9.5)	4 (8.9)	>0.999
Kidney failure, *n* (%)	0 (0)	2 (4.4)	>0.999
Ventricular tachycardia, *n* (%)	0 (0)	0 (0)	–
Cardiac rehospitalization, *n* (%)	4 (19)	8 (17.8)	>0.999
Gastrointestinal hemorrhage, *n* (%)	0 (0)	1 (2.2)	>0.999
All-cause mortality, *n* (%)	0 (0)	0 (0)	–
Mortality from other causes, *n* (%)	0 (0)	3 (6.7)	0.546
Poor medication adherence, *n* (%)	1 (4.8)	3 (6)	>0.999
Lost to follow-up, *n* (%)	1(4.8)	1(2.2)	0.538

MACCE, major adverse cardiac and cerebral events; TVR, target vessel revascularization; MI, myocardial infarction; ISR, in-stent restenosis; ELCA, excimer laser coronary angioplasty; CTO, chronic total occlusion; RA, rotational atherectomy.

* *p* < 0.05 is considered significant.

Cox proportional hazards regression models were used to assess the association between atherectomy modality (ELCA vs. RA) and clinical outcomes, with adjustment for baseline covariates (moderate/severe calcification, lesion length >20 mm, ISR-CTO, and diabetes mellitus). As detailed in Table 6, the adjusted analysis demonstrated no increased risk of MACCE with ELCA vs. RA (adjusted HR 1.53, 95% CI 0.35–6.65, *p* = 0.569). Similarly, adjusted models showed no significant differences in cardiac rehospitalization (HR 1.39, 95% CI 0.33–5.84, *p* = 0.653), recurrent angina (HR 0.78, 95% CI 0.09–6.87, *p* = 0.824), recurrent MI (*p* = 0.742), or TVR (*p* = 0.754).

**Table 6:** The hazard ratio of clinical outcomes during follow-up was compared between ELCA group and RA group.

Variable	Unadjusted			Adjusted		
	HR	*95%*CI	*p-value* *	HR	95%CI	*p-value* *
MACCE rate	1.51	0.42–5.35	0.526	1.53	0.35–6.65	0.569
TVR rate	0.90	0.17–4.66	0.901	1.33	0.23–7.81	0.754
MI rate	–	–	0.690	–	–	0.742
Stroke rate	–	–	0.435	–	–	0.850
Heart failure rate	0.77	0.08–7.45	0.824	1.13	0.11–12.14	0.921
Angina rate	1.14	0.21–6.23	0.883	0.78	0.09–6.87	0.824
Kidney failure rate	–	–	0.620	–	–	0.898
Cardiac rehospitalization rate	0.94	0.25–3.55	0.924	1.39	0.33–5.84	0.653

HR, hazard ratio; CI, confidence interval; MACCE, major adverse cardiac and cerebral events; TVR, target-vessel revascularization; MI, myocardial infarction.

* *p* < 0.05 is considered significant.

**Figure 3:**
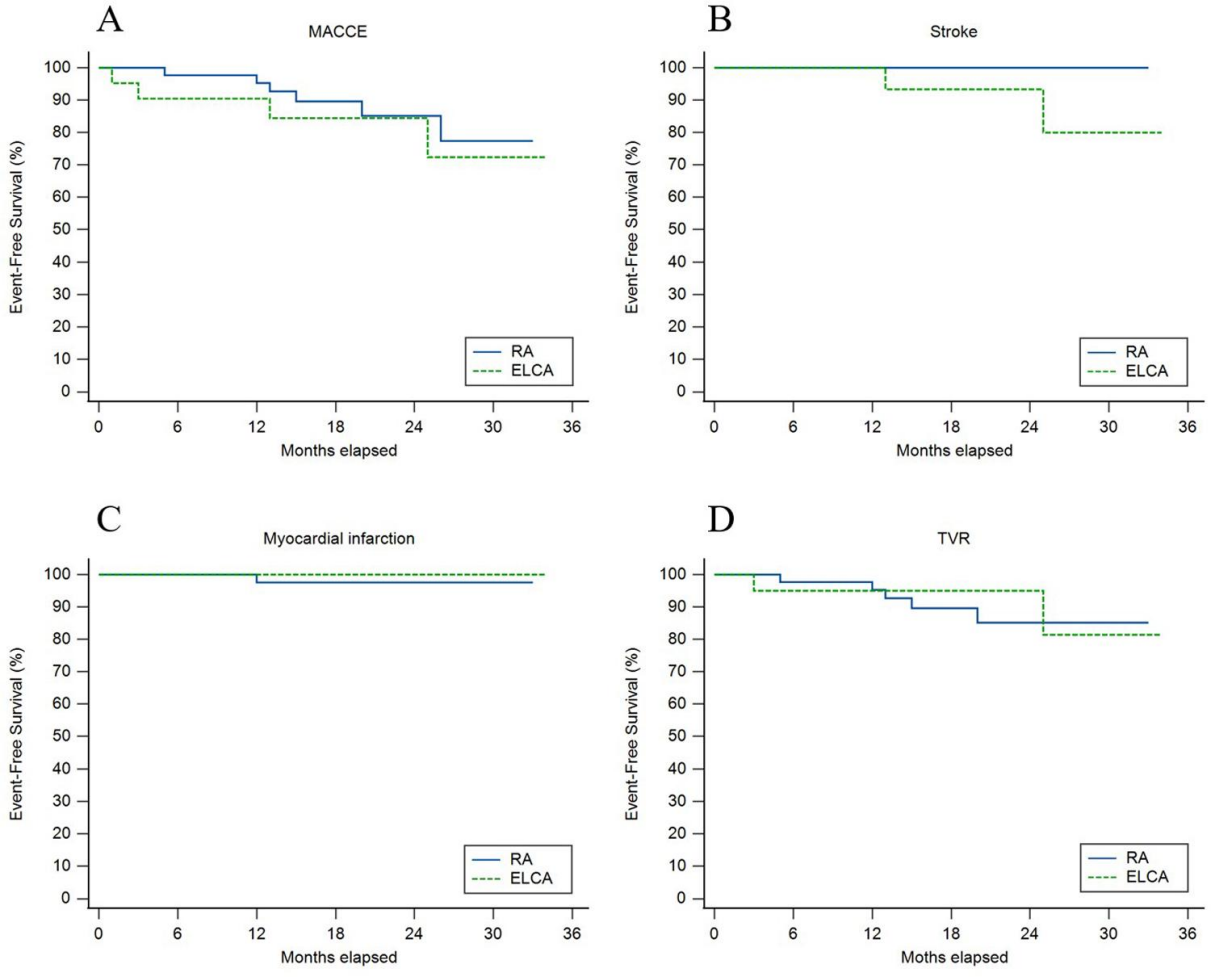
Unadjusted kaplan–meier curves for **(A)** major adverse cardiovascular and cerebrovascular events (MACCE), **(B)** stroke, **(C)** myocardial infarction, and **(D)** target vessel revascularization (TVR) between ELCA and RA groups.

Figure 3 displays unadjusted Kaplan–Meier curves comparing event-free survival between ELCA and RA after CTO-PCI. Over a median follow-up of 17.5 months (IQR 13–25.25), MACCE-free survival did not differ significantly between groups (*χ*² = 0.41, *p* = 0.523). Stroke-free survival showed a nominal difference (*χ*² = 4.12, *p* = 0.042), with two stroke events in the ELCA group (lacunar infarction at 13 months and brainstem infarction at 25 months post-PCI), both managed medically without residual deficits, vs. no strokes in the RA group. One RA patient developed target vessel-related acute inferior wall MI at 12 months (successfully treated with PCI), though MI-free survival demonstrated no intergroup difference (*χ*² = 0.46, *p* = 0.496). TVR-free survival rates declined comparably from 100% to 85.1% (ELCA) and 81.4% (RA) (*χ*² = 0.02, *p* = 0.901).

## Discussion

As the first comparative analysis of ELCA and RA for CTO-PCI in a Chinese population, our study reveals comparable procedural success rates between ELCA and RA in CTO-PCI, with RA preferentially utilized for moderate/severe calcification (*p* = 0.020), while ELCA dominated in longer lesions (*p* = 0.044) and ISR-CTO cases (*p* = 0.024). Despite complex lesion morphology (multivessel disease prevalence >60%), both techniques showed similar safety profiles, including in-hospital complications, stroke, MI, MACCE, and long-term survival, both confirmed by Kaplan–Meier analysis (Figure 3) and adjusted Cox models (Table 6).

In CTO-PCI, balloon uncrossable and undilatable lesions are challenging, accounting for 9% and 2% of failures, respectively, often due to severe calcification or poor plaque compliance (14). Both RA and ELCA are widely used to address these issues, with RA being the primary tool for debulking calcified lesions, whereas ELCA is particularly efficacious in treating device-uncrossable CTO lesions. However, each technique has distinct complication profiles: The higher incidence of transient slow/no-reflow with ELCA (12% vs. 0%, *p* = 0.061) may be partially attributable to its photomechanical mechanism: ultraviolet laser-induced acoustic waves fragment plaque into microparticles, with inadequate saline flushing potentially contributing to microvascular embolization (15), while RA incurred two perforations (4%) potentially attributable to severe calcification (76% vs. 48% in ELCA). Our RA outcomes align with Safian et al.'s evidence that smaller burrs (1.25–1.50 mm in 98% of cases) reduce vascular complications vs. larger burrs (5.1% vs. 12.7%, *p* < 0.05) (16). Although no risk stratification using the Thrombolysis in Myocardial Infarction Risk Score for Secondary Prevention (TRS 2°P) was performed (17), all patients received standardized post-procedural dual antiplatelet therapy (DAPT), and Both techniques achieved benchmark success rates (RA 96% vs. ELCA 88%), consistent with Juan et al.'s multicenter ELCA data (91% overall success) (18) and French registry RA outcomes (19). These findings validate lesion-specific utility—RA for calcified niches, ELCA for ISR-CTO/long lesions requiring wire preservation—while emphasizing proactive risk mitigation (saline flushing for ELCA, burr-size optimization for RA) (20, 21). In clinical practice, selecting the appropriate technique based on lesion characteristics and optimizing procedural strategies can enhance procedural safety and efficacy.

Prior studies have consistently demonstrated that plaque modification techniques prolong procedural metrics in CTO-PCI. Karacsonyi et al. reported significantly longer procedure times (169 vs. 130 min) and fluoroscopy times (64 vs. 50 min) with ELCA in 752 CTO lesions (5). Similarly, Ayoub et al. observed that RA extended procedure duration (127 vs. 81 min), fluoroscopy time (54 vs. 35 min), and radiation dose (12,881 vs. 9,710 cGy·cm², *p* < 0.001) compared to non-RA cases (22). In our cohort, both ELCA and RA increased procedural duration and radiation exposure compared to conventional PCI—a pattern consistent with prior studies (23)—though inter-device differences were non-significant (*p* > 0.05). This aligns with the inherent technical complexity of atherectomy, requiring meticulous lesion preparation under prolonged imaging guidance.

In China, RA and ELCA currently rely on imported devices, resulting in high equipment costs. In our study, laser catheters accounted for 46.87% of ELCA procedural costs, while RA burr systems represented 26.82% of total expenses. Although ELCA's overall procedural cost exceeded RA, device-excluded costs showed no significant difference (*p* = 0.210). Current challenges stem from limited reimbursement coverage for atherectomy under China's national health insurance system and regional policy disparities. Future cost reductions through device localization​ [prioritized in China's “14th Five-Year Plan” (24)]—a critical concern for most hospitals globally as evidenced by international comparative cost analyses (25)—and optimized reimbursement policies​ could expand accessibility, enabling broader clinical adoption to benefit patients.

During a median follow-up of 17.5 months, both ELCA and RA demonstrated comparable rates of TVR (9.5% vs. 11.1%, *p* = 0.754) and MACCE (19% vs. 13.3%, *p* = 0.569), with no cardiac deaths observed. These findings align with Ojeda et al.'s multicenter ELCA study (TVR 11.9% at 14 months) (26), confirming the long-term safety of both techniques. Notably, two stroke events occurred in the ELCA group (9.5% vs. 0%, *p* = 0.098): one lacunar infarction in a noncompliant smoker at 2 months post-PCI, and one brainstem infarction at 25 months without residual deficits. Both cases were adjudicated as unrelated to the procedure, consistent with prior ELCA studies showing no increased late stroke risk (27). Cox regression adjusting for key anatomical and metabolic confounders (moderate/severe calcification, lesion length >20 mm, ISR-CTO, diabetes mellitus) revealed no MACCE difference between ELCA and RA (adjusted HR 1.53, 95% CI 0.35–6.65, *p* = 0.569), mirroring independent reports that neither RA nor ELCA independently elevate MACCE risk (28, 29).

This study provides the first comparative analysis of ELCA and RA in CTO percutaneous coronary intervention, specifically assessing procedural success, complication rates, and mid-term outcomes. A major strength lies in its focus on device-uncrossable CTOs with ambiguous true lumen confirmation, demonstrating that both techniques can be performed with acceptable and controllable risks even in this high-risk setting. Additionally, the inclusion of a comparative cost analysis offers valuable practical insights for institutions adopting these techniques for CTO revascularization.

Several limitations warrant consideration. First, the single-center design and modest sample size (*n* = 75) may limit generalizability. *post-hoc* power analysis indicated insufficient statistical power (8.7%) to detect the observed 5.7% MACCE rate difference between ELCA and RA (19% vs. 13.3%), calculated using Cohen's h = 0.128 under α=0.05 two-tailed assumptions. While this raises concerns about Type II error, the non-significant *p*-value (0.569) and outcome rates aligning with multinational registries suggest clinical equipoise (4, 30). Second, non-randomized allocation introduced selection bias, as evidenced by RA's preferential use in calcified lesions (76% vs. 48%, *p* = 0.020). Although we adjusted for calcification severity in Cox models, unmeasured confounders (e.g., operator experience) may persist. Finally, the median 17.5-month follow-up precludes assessment of very late stent thrombosis—a critical endpoint requiring extended surveillance in CTO studies (31).

## Conclusion

In this Chinese cohort, both ELCA and RA demonstrate comparable procedural success (88%–96%) and mid-term safety in balloon-uncrossable CTO lesions. ELCA provides a safe and effective alternative when RotaWire™ exchange is not feasible after successful CTO guidewire crossing, particularly for ISR-CTO or long lesions (>20 mm). Device selection should balance lesion morphology, institutional expertise, and cost considerations.

## Data Availability

The original contributions presented in the study are included in the article/Supplementary Material, further inquiries can be directed to the corresponding authors.
